# Stepwise Assessment of Computational Coronary Physiology and Plaque Vulnerability: Impact on Coronary Revascularization Decision Making

**DOI:** 10.1016/j.jacasi.2025.08.015

**Published:** 2025-12-11

**Authors:** Chenguang Li, Daixin Ding, Zhiqing Wang, Yong He, Yong Dong, Junqing Yang, Zheng Shen, Defeng Pan, William Wijns, Junbo Ge, Shengxian Tu

**Affiliations:** a Department of Cardiology, Zhongshan Hospital, Fudan University, National Clinical Research Center for Interventional Medicine, Shanghai Clinical Research Center for Interventional Medicine, Shanghai, China; b Department of Cardiology, Renji Hospital, School of Medicine, Shanghai Jiao Tong University, Shanghai, China; c Department of Cardiology, Huadong Hospital, Fudan University, Shanghai, China; d Department of Cardiology, West China Hospital Sichuan University, Chengdu, China; e Department of Cardiology, The 7th People’s Hospital of Zhengzhou, Zhengzhou, China; f Department of Cardiovascular Medicine, Guangdong Cardiovascular Institute, Guangdong Provincial People's Hospital, and Guangdong Academy of Medical Sciences, Guangzhou, China; g The Affiliated Hospital of Guizhou Medical University, Guizhou, China; h Department of Cardiology, Affiliated Hospital of Xuzhou Medical University, Jiangsu, China; i Lambe Institute for Translational Research, Smart Sensors Laboratory and CÚRAM, University of Galway, Galway, Ireland; j Department of Cardiology, Ren Ji Hospital, School of Medicine, and School of Biomedical Engineering, Shanghai Jiao Tong University, Shanghai, China

**Keywords:** coronary angiography, optical coherence tomography, quantitative flow ratio, radial wall strain, revascularization decision making

## Abstract

**Background:**

Incremental information on coronary physiology and plaque vulnerability may improve risk stratification beyond anatomy. Murray law–based quantitative flow ratio (μFR) and radial wall strain (RWS) are angiography-derived indices for assessing coronary physiology and plaque vulnerability, but their impact on revascularization decisions remains unclear.

**Objectives:**

The authors aimed to evaluate the impact of incremental availability of μFR and RWS on revascularization decisions.

**Methods:**

A web-based survey was conducted, comprising 25 angiographically intermediate lesions. Data from μFR, RWS, and optical coherence tomography (OCT) were stepwisely available to participating cardiologists to make revascularization decisions (medical therapy alone or revascularization) for each lesion: Decision I was made based on angiography and clinical data, Decision II was made after μFR was disclosed, Decision III followed after RWS disclosure, and Decision IV followed after OCT disclosure.

**Results:**

A total of 87 interventional cardiologists from 30 Chinese clinical centers provided 1,975 lesion-based decision sets. Following stepwise data disclosure, revascularization decisions remained unchanged in 1,013 (51.3%) decision sets. From Decision I to Decision II, 416 (21.1%) treatment recommendations changed, with 322 shifting from revascularization to medical therapy. From Decision II to Decision III, 315 (15.9%) recommendations changed, with 223 from medical therapy to revascularization. From Decision III to Decision IV, 564 (28.6%) recommendations changed, with 526 from medical therapy to revascularization. If decisions were strictly based on OCT-derived lipid-to-cap ratio, a validated quantification of plaque vulnerability, only 317 (16.1%) decisions would change from Decision III to Decision IV.

**Conclusions:**

Revascularization decisions for intermediate lesions changed significantly with sequential diagnostic data. Adding μFR to angiography decreased revascularization rates. Adding plaque vulnerability assessment significantly increased revascularization rates.

Fractional flow reserve (FFR)-guided percutaneous coronary intervention (PCI) has established its clinical superiority over angiography-guided PCI alone in managing ischemia-inducing coronary stenoses.^1^ Despite the proven safety and efficacy of FFR-guided PCI, a subset of non–flow-limiting lesions continues to contribute to major adverse cardiac events, mainly due to atherosclerotic progression or plaque destabilization in angiographically intermediate lesions managed conservatively.^2^ Identifying these vulnerable plaques could be facilitated by intracoronary imaging such as optical coherence tomography (OCT) or intravascular ultrasound (IVUS).

The recent PREVENT (Preventive percutaneous coronary intervention versus optimal medical therapy alone for the treatment of vulnerable atherosclerotic coronary plaques [PREVENT]: a multicentre, open-label, randomised controlled trial) showed that preventive PCI in non–flow-limiting lesions with OCT-detected vulnerable plaques significantly reduced adverse cardiac events compared with medical therapy alone.^3^ These findings underscore the importance of incorporating plaque vulnerability assessment alongside coronary physiology to improve risk stratification, particularly in angiographically intermediate lesions, which pose unique clinical challenges. Current guidelines recommend the combined use of OCT and FFR for optimal diagnosis and tailored treatment strategies.^4^ However, the clinical use of both OCT and FFR remain limited, let alone their systematic integration into routine practice.^2^^,^^5^^,^^6^

Quantitative flow ratio (QFR) and radial wall strain (RWS) are novel angiography-derived computational indices for assessing coronary physiology and plaque vulnerability, respectively, without the need for additional invasive instrumentation or vasodilators. QFR demonstrates 86% to 93% diagnostic concordance with FFR for identifying ischemia-causing stenosis,^7^^,^^8^ and a recent randomized controlled trial confirmed the superior clinical utility of QFR over angiography in guiding PCI.^9^ RWS has been shown to correlate positively with OCT-derived vulnerable plaque characteristics,^10^ including thin-cap fibroatheroma (TCFA) and lipid-to-cap ratio (LCR)—a novel index of plaque vulnerability capable of identifying event-causing, nonculprit lesions.^11^ Moreover, the combination of RWS on top of QFR provides superior discrimination of non–flow-limiting vessels that can be safely deferred.^12^

The QFR method was newly upgraded to incorporate Murray bifurcation fractal law, which allows FFR computation from a single angiographic view.^13^ This Murray law–based quantitative flow ratio (μFR) has shown improved feasibility and comparable diagnostic accuracy to traditional QFR.^14^^,^^15^ The routine availability of μFR and RWS for assessing intermediate stenosis has the potential to optimize clinical management by integrating ischemic burden and plaque vulnerability; however, the extent to which the incremental availability of μFR and RWS would influence clinical decision making remains unknown. In addition, whether OCT-derived information would further modify revascularization decision making remains to be explored.

This survey was launched to evaluate the influence of incremental information regarding coronary physiology and plaque vulnerability on revascularization decision making for treatment of intermediate coronary stenosis. Furthermore, we examined how interventional experience of the cardiologists affects decision-making patterns. Here, we present the key findings of this survey investigation.

## Methods

### Objective and hypothesis

This survey was designed to investigate the influence of incremental information on revascularization strategy making for the management of intermediate coronary stenosis. We hypothesized that revascularization decisions based on invasive coronary angiography (ICA) alone would be significantly altered by the addition of μFR and RWS and the further incorporation of OCT data would not lead to substantial modifications in decision making.

### Overall survey design

**Figure 1:**
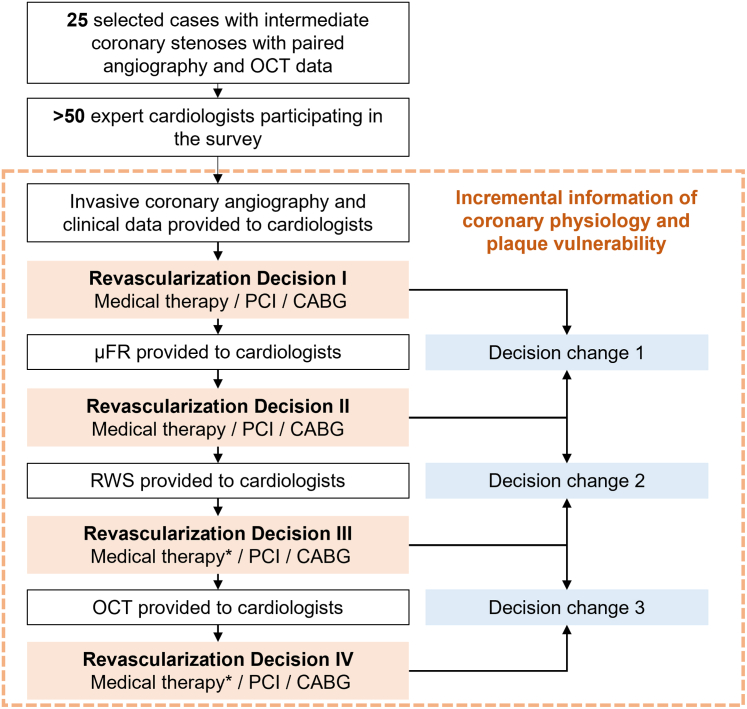
Survey Design A web-based survey was designed to investigate the impact of incremental information of coronary physiology and plaque vulnerability on revascularization decision making. ∗Classified as either standard medical therapy or intensive medical therapy. CABG = coronary artery bypass grafting; OCT = optical coherence tomography; PCI = percutaneous coronary intervention; RWS = radial wall strain; μFR = Murray law–based quantitative flow ratio.

The survey was hosted on a dedicated website that could be accessed by participating cardiologists via a secure link between October 1, 2024, and December 23, 2024. It comprised 2 separate parts, namely structured questions and case-based assessments. In the first part, participants were asked to describe their experience with PCI, including age; years of experience in interventional cardiology; annual PCI volume; and proficiency with techniques including FFR, μFR, IVUS, OCT, and RWS (Supplemental Table 1). The survey used predefined categories and single-choice format. In the second part, cardiologists were asked to make revascularization decisions for angiographically intermediate stenoses, based on incremental information provided in a stepwise order: ICA, μFR, RWS, and OCT (Figure 1). OCT-derived LCR was available for post hoc comparison but never disclosed to participants. For each lesion, a set of 4 consecutive revascularization decisions were recorded following the stepwise data disclosure. Participants were requested to make decisions under simulated ideal clinical conditions, focusing exclusively on optimal clinical practice in this virtual catheterization laboratory, without consideration of financial constraints or local regulations. Participants were informed of the recommended cutoff values for μFR and RWS: μFR ≤0.80 for identifying physiologically significant stenosis^4^ and RWS ≥13.0% for identifying vulnerable plaque.^12^ For a response to be considered valid, all 4 revascularization decisions had to be completed and recorded. Once a decision was submitted, it could not be altered and participants could only proceed to the next case on completion of the ongoing one. Access to the preceding data was restricted to interventional cardiologists, whose identities were verified before participation. Participants were informed about the survey objectives and provided their agreement by responding positively to the invitation.

### Stepwise data presentation and revascularization decision making

**Figure 2:**
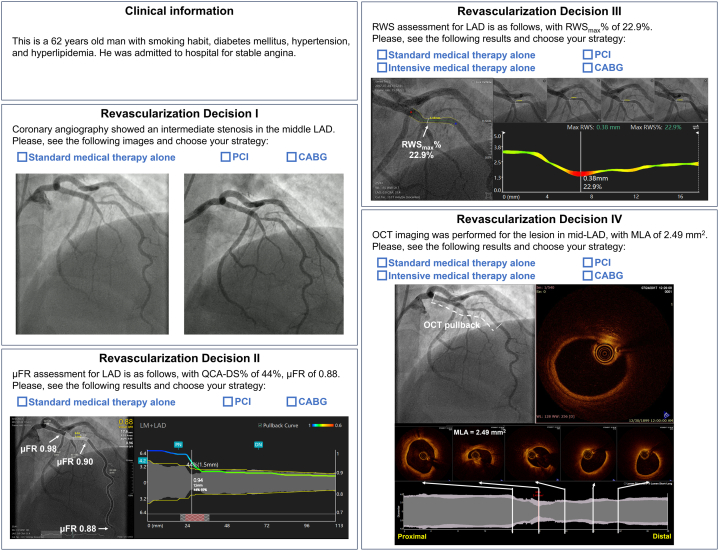
Case Example of Revascularization Decision Making Participants were asked to make revascularization decisions for intermediate stenoses based on incremental information. The first revascularization decision (Decision I) was made when baseline patient characteristics and 2 optimal angiographic projections of the interrogated lesion were available. μFR, RWS, and OCT data were not disclosed at this stage. The second revascularization decision (Decision II) was made when μFR data were disclosed. RWS and OCT data were not disclosed at this stage. The third revascularization decision (Decision III) was made when RWS data were further disclosed. The percent lumen diameter change of each location along the lesion was visualized as a color-coded curve, with the hot spot(s) indicating the location(s) with largest lumen deformation suspect of vulnerable plaque. OCT data were not disclosed at this stage. The fourth revascularization decision (Decision IV) was made when OCT data were finally provided as high-resolution videos. DS% = percent diameter stenosis; ICA = invasive coronary angiography; LAD = left anterior descending artery; MLA = minimal lumen area; QCA = quantitative coronary angiography; other abbreviations as in Figure 1.

In the second part of the survey, cases were selected by an independent interventional cardiologist (Z.W.) from a database at an academic core laboratory (CardHemo, Shanghai Jiao Tong University), which was composed of 2 independent datasets with available ICA, ICA-based computational indices (μFR and RWS), and OCT data.^10^^,^^16^ The local ethics committee or institutional review board at each participating center approved the collection and potential future use of the data for research purposes and all patients provided written informed consent. In this database, patients presenting with stable angina or non–ST-segment elevation acute coronary syndrome were screened. Eligible lesions in epicardial coronary arteries should have a visually estimated diameter stenosis of 40% to 70%^17^ and a reference lumen diameter of >2.25 mm. Chronic total occlusion, left main disease, diffuse small-vessel disease, and stenoses identified as the culprit for patient’s symptoms were excluded. Finally, a total of 25 angiographically intermediate coronary lesions in 25 eligible patients were selected. Among these interrogated lesions, 7 were functionally significant (μFR ≤0.80),^9^ 10 were RWS positive (RWS ≥13.0%),^10^^,^^12^ and 10 were LCR positive (LCR >0.33).^11^ Baseline patient and lesion characteristics are shown in Supplemental Table 2. For each lesion, 4 sequential revascularization decisions were recorded, following a structured stepwise approach (Figure 2):

1. Decision I (ICA-based)

First, baseline patient and vessel characteristics and 2 optimal angiographic projections were available to the participants to make their first revascularization decision (Decision I), choosing one of the following options: 1) standard medical therapy alone, not requiring PCI or coronary artery bypass grafting (CABG); 2) PCI; or 3) CABG. Angiograms were provided as high-resolution videos. Participants could pause, scroll frame-by-frame, or zoom-in and zoom-out, mimicking real catheterization laboratory or heart team conditions. Importantly, μFR, RWS, and OCT data were not disclosed to participants at this stage.

2. Decision II (ICA+μFR-based)

Next, μFR data were further disclosed to the participants to inform a second revascularization decision (Decision II), choosing from the same 3 candidate options. The μFR data included a screenshot of the virtual pressure pullback curve superimposed on the interrogated artery, displaying the physiological disease pattern.^18^ The focal μFR values were given at the proximal lesion edge, distal lesion edge, and distal vessel, allowing assessment of the pressure gradient within and outside the lesion. The quantitative coronary angiography data including minimum lumen diameter and percent diameter stenosis were simultaneously provided. Importantly, RWS and OCT data were not disclosed at this stage.

3. Decision III (ICA+μFR+RWS-based)

Third, RWS data were revealed for making a third revascularization decision (Decision III). Because RWS is an index assessing plaque vulnerability and intensive lipid-lowering therapy for vulnerable plaque has been proven effective for reducing adverse cardiovascular events,^19^ a new option was added, namely “intensive medical therapy, not requiring PCI or CABG.” RWS data were provided as screenshots side by side with μFR data. The percent lumen diameter change of each location along the lesion was visualized as a color-coded curve, with the hot spots indicating the sites with high strain pattern indicative of vulnerable plaques. OCT data were not yet disclosed at this stage.

4. Decision IV (ICA+μFR+RWS+OCT-based)

Finally, original OCT pullback together with minimal lumen area (MLA) data were disclosed for making the final revascularization decision (Decision IV), selecting from the same 4 options as for Decision III. The OCT data were shown as high-resolution videos. The start and end locations of the OCT pullback were coregistered with the corresponding angiographic images. The longitudinal profile of the reconstructed lumen segment imaged by OCT was also provided. The frame with MLA and 4 other representative frames of plaque features were provided, with their respective locations marked on the longitudinal profile.

All images were anonymized and analyzed at the CardHemo core laboratory (Shanghai Jiao Tong University). One-stop-shop μFR-RWS assessment and OCT image analysis were performed by certified analysts using the AngioPlus Core software (Version V3, Pulse Medical) and the OctPlus software (Version V2, Pulse Medical), respectively. Cases were randomly assigned, with all participants following the same case sequence during analysis.

### Study endpoints

On survey completion, for each lesion, a set of 4 revascularization decisions was recorded: Decision I (ICA-based), Decision II (ICA+μFR-based), Decision III (ICA+μFR+RWS-based), and Decision IV (ICA+μFR+RWS+OCT-based). A shift in revascularization recommendations was identified as: 1) a shift from medical therapy alone, including both standard and intensive medical therapy, to revascularization (PCI or CABG); or 2) a shift from revascularization to medical therapy alone.

The primary endpoint was defined as the proportion of cases in which the ICA-based revascularization decision changed after the disclosure of incremental data, analyzed in 3 steps: 1) proportion of cases after μFR data disclosure; 2) proportion of cases after RWS data disclosure; and 3) proportion of cases after qualitative OCT data disclosure. To explore the influence of qualitative OCT reading vs quantitative OCT-derived LCR on revascularization decisions, the proportion of cases that would change from Decision III to Decision IV if LCR had been used as the sole quantitative criterion was reported.

The secondary endpoint was to identify participants’ experience-related factors that were likely to result in more revascularization decision changes following stepwise data disclosure.

### Statistical analysis

Continuous variables were tested for normal distribution by Kolmogorov-Smirnov test and were reported as mean ± SD if normally distributed or as median (IQR) if non-normally distributed. Categorical variables were reported as counts (percentage). The difference in the pattern of 4 revascularization decisions was evaluated by using the McNemar test. Changes in decision patterns between consecutive decisions were visualized by Sankey plot. Cohen Kappa test was used to investigate the consistency in the pattern of 4 revascularization decisions. To identify predictors of revascularization decision changes with incremental data disclosure, mixed-effects logistic regression models with random effects accounting for center-level and participant-level clustering were used. The multivariate logistic regression model incorporated all participant characteristics related to experience with PCI that showed a single *p* value of <0.10 in the univariate regression model, including age (≤40 years or >40 years); overall experience in interventional cardiology (≤2 years, >2 and ≤5 years, >5 and ≤10 years, or >10 years); annual PCI volume (≤100, 101-300, 301-500, or >500/year); and proficiency in FFR, μFR, OCT, IVUS, and RWS (no experience, <1 year, 1-3 years, or >3 years). The Cochran-Armitage test was used to assess the association between the decision patterns and levels of the preceding participants’ characteristics. A 2-sided *P* value <0.05 was considered significant. Analyses were performed using Stata version 16.0 (StataCorp).

## Results

### Characteristics of the survey participants

From October 1, 2024, to December 23, 2024, 87 interventional cardiologists from 30 clinical centers across China participated in the survey. Table 1 shows the baseline characteristics of all physicians participating in the survey. The mean age of the participants was 39 ± 8 years, and 49.4% (43 of 87) had more than 10 years of experience in interventional cardiology. Most participants, 85.1% (74 of 87), reported an annual PCI volume exceeding 100 procedures. Regarding the use of invasive imaging and physiology techniques, intravascular ultrasound was most widely utilized, with 71.3% (62 of 87) of participants having more than 3 years of experience with this modality. For angiography-derived assessments, 77.0% (67 of 87) of participants reported experience with μFR, whereas only 24.1% (21 of 87) had experience with RWS. A list of the participating clinical centers is provided in Supplemental Table 3. The STROBE checklist is shown in Supplemental Table 4.

**Table 1:** Characteristics of the Survey Participants

Total number of centers	30
Total number of participants	87
Age, y	39 ± 8
Experience in interventional cardiology, y	
≤2	7 (8.0)
>2 and ≤5	16 (18.4)
>5 and ≤10	21 (24.1)
>10	43 (49.4)
Number of yearly PCIs	
≤100	13 (14.9)
101-300	27 (31.0)
301-500	24 (27.6)
>500	23 (26.4)
Experience with FFR, y	
No experience	4 (4.6)
<1	12 (13.8)
1-3	27 (31.0)
>3	44 (50.6)
Experience with μFR, y	
No experience	20 (23.0)
<1	20 (23.0)
1-3	24 (27.6)
>3	23 (26.4)
Experience with IVUS, y	
No experience	1 (1.1)
<1	5 (5.7)
1-3	19 (21.8)
>3	62 (71.3)
Experience with OCT, y	
No experience	11 (12.6)
<1	18 (20.7)
1-3	25 (28.7)
>3	33 (37.9)
Experience with RWS, y	
No experience	66 (75.9)
<1	13 (14.9)
≥1	8 (9.2)

Values are n (%) or mean ± SD.

FFR = fractional flow reserve; IVUS = intravascular ultrasound; OCT = optical coherence tomography; PCI = percutaneous coronary intervention; RWS = radial wall strain; μFR = Murray law–based quantitative flow ratio.

### Overall revascularization decision patterns

Among the 87 participants, most (79.3% [69 of 87]) completed the survey for all 25 cases, 10.3% (9 of 87) evaluated more than 10 but fewer than 25 cases, and another 10.3% (9 of 87) evaluated 10 or fewer cases. This yielded a total of 1,975 lesion-based decision sets, each comprising 4 stepwise decisions (Decision I to Decision IV). Unless otherwise specified, the results are summarized on a per-lesion basis. Supplemental Table 5 presents the distribution of Decisions I to IV across all cases. The overall revascularization rates (including PCI or CABG) were as follows: 35.1% (693 of 1,975) for Decision I, 23.5% (465 of 1,975) for Decision II, 30.2% (596 of 1,975) for Decision III, and 54.9% (1,084 of 1,975) for Decision IV. Moderate concordance was observed between consecutive decisions, with Kappa coefficients ranging from 0.45 to 0.60 (Table 2).

**Table 2:** Concordance Between Consecutive Decisions

		Medical Therapy Alone	Revascularization	Total
		Decision II		
Decision I	Medical therapy alone	1,188	94	1,282
	Revascularization	322	371	693
Total		1,510	465	1,975
Kappa coefficient = 0.50 (95% CI: 0.46-0.54), *P* < 0.0001				

		Decision III		
Decision II	Medical therapy alone	1,287	223	1,510
	Revascularization	92	373	465
Total		1,379	596	1,975
Kappa coefficient = 0.60 (95% CI: 0.55-0.64), *P* < 0.0001				

		Decision IV		
Decision III	Medical therapy alone	853	526	1,379
	Revascularization	38	558	596
Total		891	1,084	1,975
Kappa coefficient = 0.45 (95% CI: 0.41-0.49), *P* < 0.0001				

Decision I is ICA-based, Decision II is ICA+μFR-based, Decision III is ICA+μFR+RWS-based, and Decision IV is ICA+μFR+RWS+OCT-based.

ICA = invasive coronary angiography; other abbreviations as in Table 1.

### Stepwise change of revascularization decision patterns

Among 1,975 lesion-based decision sets, decisions remained consistent across all 4 steps in 51.3% (1,013 of 1,975) of cases, despite the progressive data disclosure. In the remaining 48.7% (962 of 1,975) of cases, at least one decision change occurred between consecutive steps.

The stepwise evolution of decision patterns is shown in the Central Illustration. Compared with angiogram-based Decision I, the introduction of μFR changed decisions in 21.1% (416 of 1,975) of cases (*P* < 0.0001), including 16.3% (322 of 1,975) shifting from revascularization to medical therapy alone and 4.8% (94 of 1,975) from medical therapy alone to revascularization. Subsequently, after RWS was disclosed, the revascularization decision changed further in 15.9% (315 of 1,975) of cases (*P* < 0.0001), with 11.3% (223 of 1975) changing from medical therapy alone to revascularization and 4.7% (92 of 1,975) vice versa. Finally, after OCT disclosure, the revascularization decisions changed in 28.6% (564 of 1,975) of cases (*P* < 0.0001), with most (26.6% [526 of 1975]) involving a shift from medical therapy alone to revascularization. Notably, in 64.2% (1,268 of 1,975) of cases, the final Decision IV aligned precisely with Decision I, regardless of any intermediate decision changes.

**Central Illustration:**
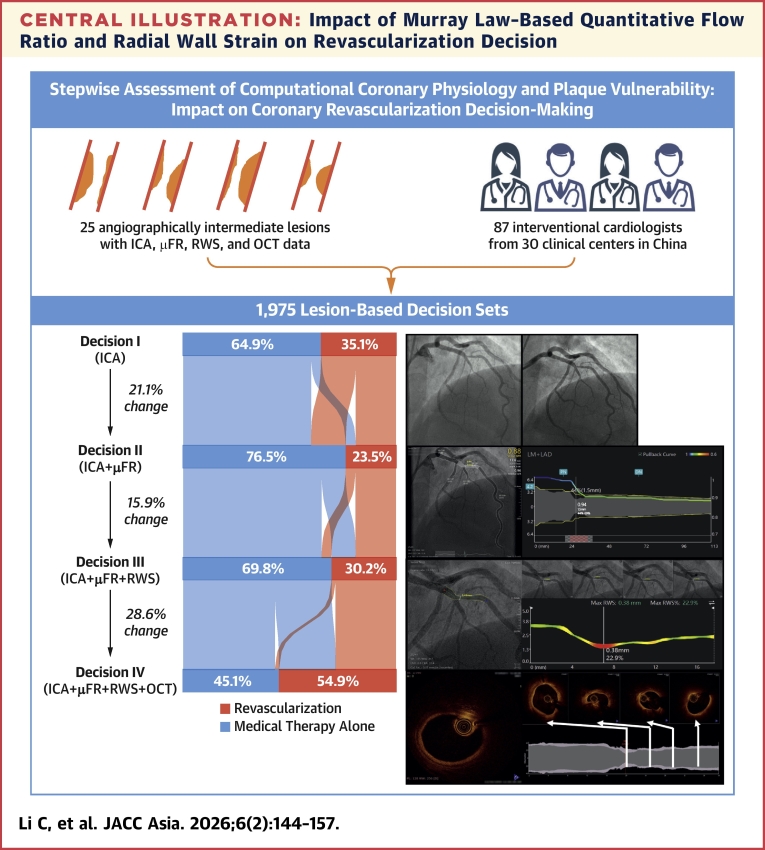
Impact of Murray Law–Based Quantitative Flow Ratio and Radial Wall Strain on Revascularization Decision This survey was designed to evaluate the impact of incremental information of coronary physiology and plaque vulnerability on revascularization decision making for angiographically intermediate stenoses. Participating cardiologists were asked to make revascularization decisions (medical therapy alone or revascularization) for each lesion based on sequential disclosure of μFR, RWS, and OCT data on top of ICA. A total of 87 interventional cardiologists from 30 clinical centers in China participated in this survey, providing 1,975 lesion-based decision sets, each comprising 4 stepwise decisions (Decision I to Decision IV). The disclosure of μFR resulted in a change of decision in 21.1% (416 of 1,975) of cases (*P* < 0.0001). After RWS was revealed, revascularization decisions changed in 15.9% (315 of 1,975) of cases (*P* < 0.0001). The decisions further changed in 28.6% (564 of 1,975) (*P* < 0.0001) after final disclosure of OCT. Overall, revascularization decision changed in 48.7% (962 of 1,975) of cases in all steps following sequential disclosure of μFR, RWS, and OCT. ICA = invasive coronary angiography; OCT = optical coherence tomography; μFR = Murray law–based quantitative flow ratio; RWS = radial wall strain.

### Influence of μFR on revascularization decision

**Figure 3:**
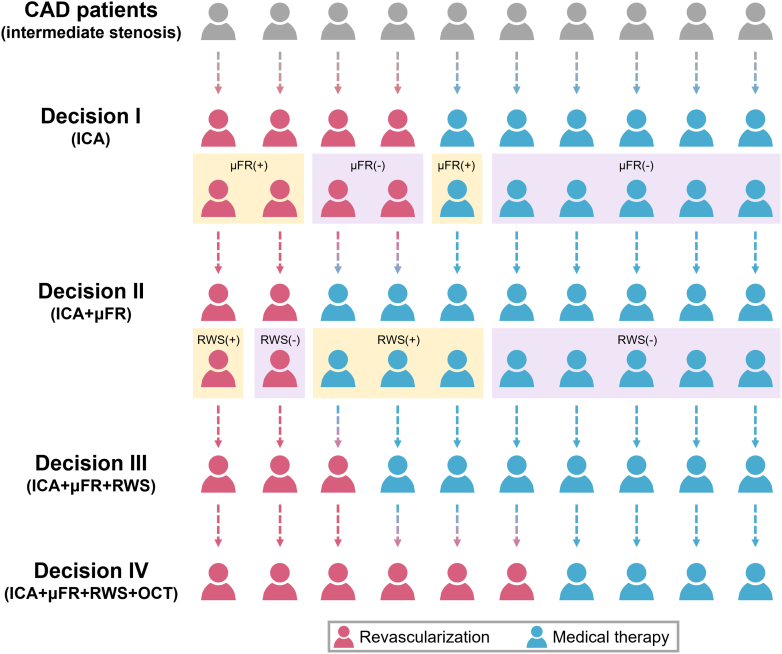
Stepwise Revascularization Decision Modifications To facilitate data interpretability, the progressive change in revascularization decisions with sequential availability of μFR, RWS, and OCT findings are visualized in a simplified schematic. Overall, the introduction of μFR reduced revascularization recommendations, mainly due to negative μFR values. Subsequent RWS disclosure increased recommendations for revascularization, driven by positive RWS findings. Final reveal of OCT further elevated revascularization recommendation rates due to positive qualitative OCT reading. CAD = coronary artery disease; other abbreviations as in Figures 1 and 2.

Among ICA-based Decision I, 31.1% (614 of 1,975) were discordant with dichotomous μFR values (Figure 3, Supplemental Figure 1): 19.3% (381 of 1,975) planned for revascularization was physiologically nonsignificant (μFR >0.80), and 11.8% (233 of 1,975) planned for medical therapy alone was physiological significant (μFR ≤0.80). Following the disclosure of μFR, 21.1% (416 of 1,975) of the initial ICA-based decisions were revised. Despite this adjustment, 15.2% (300 of 1,975) of Decision II remained discordant with μFR values: 5.6% (110 of 1,975) of cases were still planned for revascularization despite having a μFR >0.80, and 9.6% (190 of 1,975) were planned for medical therapy alone despite having a μFR ≤0.80. These discrepancies were predominantly observed in lesions within the gray zone (0.75 ≤ μFR ≤ 0.85), with a significantly higher discordance rate of 27.5% (220 of 799) compared with 6.8% (80 of 1,176) outside this range (*P* < 0.001).

### Influence of RWS on revascularization decision

Among the 1,510 (76.5%) cases initially planned for medical therapy alone in ICA+μFR-based Decision II, 566 (28.7%) cases had a positive RWS (≥13.0%), with 192 (9.7%) of these being reassigned to revascularization; the remaining 944 (47.8%) cases with a negative RWS (<13.0%) saw only 31 (1.6%) such shifts (Figure 3, Supplemental Figure 2). Comparatively, among 465 (23.5%) cases initially planned for revascularization in Decision II, 233 (11.8%) cases had a negative RWS, and 76 (3.8%) of these were shifted to medical therapy alone, whereas only 16 (0.8%) of the remaining 232 (11.7%) cases with a positive RWS underwent such changes. In addition, 855 (43.3%) cases initially assigned to standard medical therapy in Decision II were escalated to intensive medical therapy in Decision III, with 296 (15.0%) of these escalations driven by the presence of a positive RWS (Supplemental Figure 3).

### Influence of OCT on revascularization decision

The disclosure of OCT pullback data and MLA further influenced revascularization decisions in 28.6% (564 of 1,975) of cases from Decision III to Decision IV. Specifically, 26.6% (526 of 1,379) of cases shifted from medical therapy alone to revascularization, whereas 1.9% (38 of 1,975) shifted from revascularization to medical therapy alone (Figure 3, Supplemental Figure 4). In addition, 5.7% (112 of 1,975) of cases initially planned for standard medical therapy in Decision III were escalated to intensive medical therapy in Decision IV (Supplemental Figure 3). Notably, if Decision IV had been strictly guided by LCR rather than qualitative OCT interpretation, the RWS-based Decision III would have been altered in only 16.1% (317 of 1,975) of cases (Supplemental Figure 4).

### Factors influencing decision changes following stepwise data disclosure

The frequency of decision changes varied significantly based on participant characteristics (Table 3). Decision change after the incorporation of μFR to ICA was not significantly affected by the interventional experience-related factors. By contrast, the proportion of decision changes following RWS disclosure was likely to decrease with increasing annual PCI volume (*P* for trend = 0.039) and greater expertise in intravascular imaging, particularly OCT (*P* for trend <0.001). These factors, together with experience in interventional cardiology and familiarity with FFR, also affected the proportion of decision change after OCT data disclosure, with increased expertise generally leading to fewer decision changes (all *P* for trend values < 0.05). In the multivariate analysis, participants with increasing experience in interventional cardiology were less likely to alter revascularization decisions with incremental data disclosure (OR: 0.39; 95% CI: 0.17-0.90; *P* = 0.03) (Supplemental Table 6).

**Table 3:** Percentage of Decision Change Varied by Levels of Participants’ Characteristics

	Levels of Experience^a^	Age, y	Overall Interventional Experience	Annual PCI Volume	Experience With FFR	Experience With μFR	Experience With IVUS	Experience With OCT	Experience With RWS
% of change from Decision I to Decision II	No. 1	20	28	23	22	16	0	24	21
	No. 2	22	21	20	17	26	26	20	21
	No. 3	/	17	20	23	22	20	20	24
	No. 4	/	22	23	20	21	21	21	26
*P* for trend		0.415	0.432	0.631	0.893	0.276	0.615	0.764	0.275
% of change from Decision II to Decision III	No. 1	16	27	21	18	15	0	23	17
	No. 2	16	14	16	17	21	35	20	12
	No. 3	/	17	15	16	16	13	14	26
	No. 4	/	15	15	16	12	16	12	0
*P* for trend		0.853	0.057	0.039	0.373	0.069	0.006	<0.001	0.058
% of change from Decision III to Decision IV	No. 1	31	47	29	33	28	0	33	29
	No. 2	26	25	35	33	30	35	32	25
	No. 3	/	30	27	29	29	30	30	39
	No. 4	/	27	24	26	28	28	24	31
*P* for trend		0.016	0.003	0.002	0.016	0.868	0.085	0.001	0.333

Abbreviations as in Table 1.

a Level No. 1 to level No. 4 of experience correspond to the following: age (≤40 years or >40 years), overall experience in interventional cardiology (≤2 years, >2 and ≤5 years, >5 and ≤10 years, or >10 years), annual PCI volume (≤100, 101-300, 301-500, or >500), experience with techniques including FFR, μFR, OCT, and IVUS (no experience, <1 year, 1-3 years, or >3 years), and experience with RWS (no experience, <1 year, ≥1 year). The symbol “/” indicates that only two age-based levels were categorized: Level No. 1 and Level No. 2.

## Discussion

In this survey, we evaluated the impact of incremental coronary physiology and plaque vulnerability assessments on revascularization decision making for angiographically intermediate stenoses. Our findings indicate that revascularization decisions were altered in nearly half of the cases when angiography was supplemented sequentially with μFR, RWS, and OCT data (Central Illustration). The stepwise decision patterns varied at each stage: 1) μFR disclosure led to a reduction in revascularization rates; 2) RWS introduction resulted in an increase in revascularization rates, reflecting the growing consideration of plaque vulnerability; and 3) OCT availability further elevated revascularization rates. Notably, RWS demonstrated greater consistency with OCT-derived LCR than with qualitative OCT analysis in guiding revascularization decisions, highlighting its potential as a noninvasive surrogate for plaque vulnerability. This is further supported by recent studies demonstrating that RWS not only correlates with OCT-detected vulnerability features but also predicts lesion-oriented composite endpoints, independent from OCT-detected vulnerability characteristics.^16^

### The influence of coronary physiology on revascularization decision making

In the ISIS (International Study on Infarct Survival) survey, that evaluated the alignment between guideline recommendations and interventional practice regarding angiographically intermediate stenosis, clinicians predominantly relied on visual estimation, resulting in 47% of decisions discordant with true physiological significance.^20^ Even in vessels deemed not to require FFR interrogation, a mismatch between QFR and angiographic stenosis occurred in 10% of the cases.^21^ Aligning with prior studies,^6^^,^^20^^,^^22^ our survey found that 31.1% (614 of 1,975) of angiography-based decisions were discordant with subsequently disclosed μFR values: 19.3% (381 of 1,975) of cases initially selected for revascularization were physiologically nonsignificant (ie, false positives), whereas 11.8% (233 of 1,975) of deferred cases were physiologically significant (ie, false negatives). These findings underscore the limitations of angiographic assessment alone and its potential to lead to inappropriate treatment in a substantial proportion of cases, as already emphasized in prior pooled data analyses.^23^

As expected, μFR availability altered revascularization decisions in 21.1% (416 of 1,975) of cases, primarily by reducing unnecessary interventions. However, 15.2% (300 of 1,975) of the μFR-based decisions remained discordant with actual μFR values: 5.6% (110 of 1,975) with negative μFR (>0.80) were still planned for revascularization and 9.6% (190 of 1,975) with positive μFR (≤0.80) were deferred. This discrepancy echoes findings from FAVOR III China (Angiographic quantitative flow ratio-guided coronary intervention [FAVOR III China]: a multicentre, randomised, sham-controlled trial), in which 8% of revascularization decisions did not adhere to the QFR threshold.^9^ Potential explanations include the following: 1) persistent reliance on angiographic assessment, as noted in the ERIS (The Nationwide Italian SICI-GISE Cross-Sectional ERIS Study: Evolving Routine Standards of FFR Use) and ISIS surveys;^6^^,^^20^ 2) the diagnostic challenge of the “physiological gray zone” (μFR 0.75-0.85), in which strict adherence to the ≤0.80 threshold may not consistently optimize clinical decisions^24^ (our data substantiate this observation, revealing significantly higher discordance rates within vs outside this gray zone [27.5% vs 6.8%, *P* < 0.001]; and 3) the evidence-supported concept of coronary physiology as a continuous risk predictor: high or near-normal FFR indicates favorable prognosis in which revascularization-associated risks may outweigh its benefits, whereas lower FFR correlates with increased ischemic risk, indicating progressively greater benefit from revascularization.^23^^,^^25^

### The influence of plaque vulnerability on revascularization decision making

Although ischemia remains a critical determinant for revascularization, a subset of non–flow-limiting lesions progresses and causes adverse events, mainly due to plaque instability and progression.^26^ COMBINE OCT-FFR (Combined optical coherence tomography morphologic and fractional flow reserve hemodynamic assessment of nonculprit lesions to better predict adverse event outcomes in diabetes mellitus patients: COMBINE [OCT-FFR] prospective study) demonstrated that non–flow-limiting lesions with vulnerable plaque characteristics had significantly worse clinical outcomes than those without such features.^2^ Preventive treatment for vulnerable plaques, alongside ischemia elimination, has been shown to improve clinical outcomes in few trials, highlighting the need for a dual focus on ischemia and plaque vulnerability.^2^^,^^3^^,^^26^ This inspired our evaluation of both coronary physiology and plaque vulnerability in revascularization decision making. The gradual shift from an “ischemia only” to “both ischemia and vulnerability” approach was evident in this survey: 15.9% (315 of 1,975) of μFR-based revascularization decisions changed when RWS, an angiography-based biomechanical quantification of plaque instability, was disclosed, with 11.3% (223 of 1,975) shifting from medical therapy alone to revascularization. In addition, 43.3% (855 of 1,975) of cases transitioned from standard to intensive medical therapy without altering revascularization decisions. Most participants favored intensive lipid-lowering medical therapy over revascularization for vulnerable plaques, consistent with guideline recommendations advocating stratified lipid-lowering therapy.^27^ A meta-analysis of 18 randomized trials showed greater risk reduction in cardiovascular events and mortality with intensive vs standard medical therapy (24% vs 10%).^19^ Conversely, evidence supporting preventive revascularization for vulnerable plaques remains limited^3^^,^^28^ and the optimal treatment strategy warrants further investigation.

Notably, 4.0% (79 of 1,975) of decisions remained for standard medical therapy despite positive RWS findings, reflecting cautious adoption of this novel angiography-based plaque vulnerability assessment, in the absence of a prospective outcome-based trial. In comparison, OCT imaging, with its superior resolution, is widely accepted as the gold standard for evaluating plaque vulnerability.^29^ In our survey, OCT availability increased revascularization rates to 54.9% (1,084 of 1,975), consistent with prior evidence of a higher PCI rate under intracoronary imaging guidance compared with physiology guidance.^17^^,^^30^^,^^31^ The added value of OCT lies in its ability to precisely quantify lesion morphology, such as MLA, and to detect high-risk plaque features, including large plaque burden, TCFA, and macrophage infiltration, all of which can predict future cardiovascular events, particularly in high-risk patients.^32^^,^^33^ However, single plaque features have low predictive value, and it remains unclear whether higher PCI rates with OCT guidance translate into improved clinical outcomes.^34^ It is worth noting that OCT is particularly recommended for assessing complex coronary lesions, as supported by a recent meta-analysis showing a significant reduction in the odds of cardiac mortality with OCT-guided PCI compared with angiography alone, particularly in complex lesions.^35^

We recently developed LCR, a novel OCT index combining prognostic plaque features, defined as lipidic plaque burden divided by fibrous cap thickness, enabling automated and reproducible coronary plaque assessment.^11^^,^^36^ In 604 patients with acute coronary syndrome, LCR predicted recurrent cardiovascular events in nonculprit vessels with an HR higher than that of TCFA.^11^ In our survey, if the final decision (Decision IV) was strictly guided by LCR, only 16.1% (317 of 1,975) of decision changes would have occurred, compared with 28.6% (564 of 1,975) of decision changes according to qualitative OCT reading, indicating better consistency of RWS with LCR for identifying plaque vulnerability than with qualitative OCT reading. Whether the observed consistency between RWS and LCR for objective plaque assessment could be used to target OCT examinations at more complex disease patterns, not just at focal plaque, warrants further evaluation.^10^^,^^16^

### Need for establishing a unified framework of integrating coronary physiology and plaque vulnerability

By providing an integration of coronary physiology and plaque vulnerability, the novel techniques are expected to be complementary and fit into different phases along the diagnostic and/or treatment flow for intermediate coronary stenosis. This is particularly relevant in the light of FAVOR III Europe (Quantitative flow ratio versus fractional flow reserve for coronary revascularisation guidance [FAVOR III Europe]: a multicentre, randomised, non-inferiority trial) findings, in which a QFR-based diagnostic strategy failed to yield a noninferior 1-year clinical outcome compared with an FFR-based strategy.^37^ The observed outcome difference might be driven by a significantly higher revascularization rate in the QFR-guided arm (54.5% vs 45.8%; *P* = 0.0001) resulting from lower QFR values (median 0.81 vs 0.84). The QFR-FFR discrepancy might have been stemmed from the semiautomatic nature of the first-generation QFR, in which manual adjustments for lumen contour and reference vessel sizing could introduce operator-dependent variability—evidenced by a 9.4% interobserver coefficient of variation in QREP (Reproducibility of quantitative flow ratio: The QREP study).^38^ In addition, although the trial populations were similar, the event rate in the FFR-guided group (4.2%) was lower than that in the DEFINE-FLAIR^39^ (Functional Lesion Assessment of Intermediate Stenosis to Guide Revascularisation) (7.0%) or iFR-SWEDHEART^40^ (Instantaneous Wave-free Ratio Versus Fractional Flow Reserve in Patients With Stable Angina Pectoris or Acute Coronary Syndrome) (6.1%) trials. A closer examination of the primary endpoint components revealed comparable rates of cardiac death (0.8% [8 of 1,008] vs 0.8% [8 of 992]) and ischemia-driven target-vessel revascularization (2.4% [24 of 1,008] vs 2.2% [21 of 992]) between QFR- and FFR-guided strategies. Because QFR is still emerging and operators were unblinded in this non–sham-controlled study, it is possible that some unplanned revascularization decisions were influenced by perceived deferral risks rather than definitive evidence of ischemia.

Notably, recent advances in μFR technology may address some of these limitations. The integration of AI in μFR has enhanced automation and reduced average analysis time to 1 minute, potentially mitigating the learning curve problems of QFR.^13^ Both inter- (0.00 ± 0.03) and intraobserver (0.00 ± 0.03) variability was low for μFR.^13^ The inclusion of the bifurcation fractal law might further improve the performance of μFR in bifurcation lesions.^41^ Beyond coronary physiological assessment, the current study shows that revascularization decisions further evolved with the disclosure of plaque vulnerability data. Pending further prospective validation, this suggests that incorporating newer-generation QFR (ie, μFR) with a systematic, stepwise approach incorporating RWS, may optimize PCI candidate selection.

Nevertheless, the frequent stepwise decision changes observed in 48.7% (962 of 1,975) of cases—often reverting to the original angiogram-based pattern—suggest potential challenges in integrating these indexes into routine practice. Specifically, discordance between indexes (eg, negative μFR indicating preserved physiology, positive RWS indicating plaque vulnerability) may have introduced decision-making conflicts. Besides, clinicians may prioritize different indexes based on individual experience or institutional protocols. These findings strongly highlight the urgent need for a unified, evidence-based framework to optimize the integration of these techniques into clinical decision making, which should ideally be established through large-scale randomized controlled trials.

### Study limitations

First, follow-up data were not available in this study, and thus the impact of μFR and RWS-guided decision making on clinical outcomes could not be assessed. Future prospective randomized trials are needed to validate the clinical utility and economic viability of this approach. Second, sample size calculation was not performed for this exploratory survey, and results with statistical significance should be interpreted with caution. Gender data were not collected from survey participants, precluding analysis of potential gender-related differences in revascularization decision patterns. Third, the data were selected only from cases with complete datasets including ICA, μFR, RWS, and OCT, thus selection bias in putting the study population together cannot be excluded. Fourth, information regarding patients’ symptoms, such as exercise tolerance limit, was not provided to the participants. This survey focused on the impact of functional indices on clinical decision making, and additional lesion characteristics that might influence decision making were not investigated, including lesion location, lesion length, and plaque characteristics. Although clinicians had access to μFR pullback curves for visual assessment of the physiological pattern of disease distribution, this study did not incorporate established quantitative analysis of physiological disease patterns (eg, pressure pullback gradient).^42^ Future studies should evaluate whether such quantitative characterization of the spatial extent and distribution of coronary artery disease could further change revascularization decision making and improve clinical outcomes.

## Conclusions

This survey found that revascularization decisions for intermediate coronary lesions were frequently modified when sequential diagnostic information was incorporated. When coronary physiological assessment by μFR was added to angiographic data, revascularization rates decreased by 11.6%. By incorporating plaque vulnerability assessment by RWS on top of coronary angiography and physiology, the revascularization rates increased by 6.7%. An ultimate disclosure of qualitative OCT findings further increased the rates of revascularization by 24.7%.

## Funding Support and Author Disclosures

This work was supported by the National Natural Science Foundation of China (82327808; 82020108015) to Dr Tu, the Science Foundation Ireland Research Professorship Award (15/RP/2765) to Dr Wijns, the China Postdoctoral Science Foundation (2024M752017) and Shanghai Magnolia Talent Program Pujiang Project (24PJD059) to Dr Ding, and was financially supported by the National Natural Science Foundation of China (82470340), Shanghai Innovative Medical Device Application Demonstration Project (23SHS04900, 24SF1901900) to Dr Li. Dr Wijns is senior advisor of Rede Optimus Research and cofounder of Argonauts, an innovation facilitator. Dr Tu is the cofounder of Pulse Medical and has received research grants and consultancy from Pulse Medical. All other authors have reported that they have no relationships relevant to the contents of this paper to disclose.
