# Supplementary material for: Stepwise Assessment of Computational Coronary Physiology and Plaque Vulnerability: Impact on Coronary Revascularization Decision Making
Source: JACC Asia. 2025 Dec 11;6(2):144–57. doi: 10.1016/j.jacasi.2025.08.015 (PMC12904827; doi:10.1016/j.jacasi.2025.08.015)
Supplement: Supplementary Tables 1-6 and Supplementary Figures 1-4 [file mmc1.docx]

**Supplemental Material for**

**Stepwise assessment of computational coronary physiology and plaque vulnerability: impact on coronary revascularization decision-making**

**Content**

[**Supplemental Table 1.** Survey part I 2](#_Toc203859137)

[**Supplemental Table 2.** Baseline patient and lesion characteristics 3](#_Toc203859138)

[**Supplemental Table 3.** List of participating centers 6](#_Toc203859139)

[**Supplemental Table 4.** STROBE checklist 8](#_Toc203859140)

[**Supplemental Table 5.** Overall patterns of Decision I to Decision IV 10](#_Toc203859141)

[**Supplemental Table 6.** Predictors of revascularization decision change 13](#_Toc203859142)

[**Supplemental Figure 1.** Influence of μFR on revascularization decision making 15](#_Toc203859143)

[**Supplemental Figure 2.** Influence of RWS on revascularization decision making 16](#_Toc203859144)

[**Supplemental Figure 3.** Step-wise change of revascularization decision patterns 17](#_Toc203859145)

[**Supplemental Figure 4.** Influence of OCT on revascularization decision making 19](#_Toc203859146)

# **Supplemental Table 1.** Survey part I

| **Question** | **Answer (tick the box that applies)** | | | |
| --- | --- | --- | --- | --- |
| Age | Please fill in _____________ | | | |
| Overall experience in interventional practice | □≤2 years | □>2 and ≤5 years | □>5 and ≤10 years | □>10 years |
| No. of yearly PCI | □≤100/year | □101-300/year | □301-500/year | □>500/year |
| Experience with FFR | □no | □<1 year | □1-3 years | □>3 years |
| Experience with μFR | □no | □<1 year | □1-3 years | □>3 years |
| Experience with IVUS | □no | □<1 year | □1-3 years | □>3 years |
| Experience with OCT | □no | □<1 year | □1-3 years | □>3 years |
| Experience with RWS | □no | □<1 year | □≥1 year |  |

FFR, fractional flow reserve; IVUS, intravascular ultrasound; OCT, optical coherence tomography; PCI, percutaneous coronary intervention; RWS, radial wall strain; μFR, Murray law-based quantitative flow ratio.

# **Supplemental Table 2.** Baseline patient and lesion characteristics

| **No.** | **Vessel** | **QCA-DS%** | **μFR** | **RWS_max_, %** | **OCT-MLA, mm^2^** | **LCR** | **Male** | **Age, years** | **BMI, kg/m^2^** | **Diabetes** | **Smoking^*^** | **Hypercholesterolemia** | **Hypertension** | **Prior ACS** | **Prior PCI** | **Prior CABG** | **Clinical presentation** |
| --- | --- | --- | --- | --- | --- | --- | --- | --- | --- | --- | --- | --- | --- | --- | --- | --- | --- |
| 1 | RCA | 49 | 0.82 | 19.6 | 1.25 | 0.80 | 1 | 67 | 24.8 | 1 | 1 | 1 | 0 | 0 | 0 | 0 | Unstable angina |
| 2 | LAD | 47 | 0.81 | 10.1 | 1.78 | 0.59 | 1 | 83 | 26.1 | 1 | 0 | 1 | 1 | 0 | 0 | 0 | AMI |
| 3 | RCA | 42 | 0.91 | 13.7 | 2.32 | 0.59 | 0 | 70 | 33.8 | 1 | 0 | 0 | 0 | 0 | 0 | 0 | Stable angina |
| 4 | RCA | 34 | 0.94 | 20.9 | 1.95 | 0.47 | 0 | 59 | 29.3 | 1 | 1 | 1 | 1 | 1 | 1 | 0 | Unstable angina |
| 5 | RCA | 46 | 0.92 | 9.3 | 2.78 | 0.09 | 1 | 63 | 29.0 | 1 | 1 | 1 | 0 | 0 | 0 | 0 | AMI |
| 6 | RCA | 45 | 0.85 | 21.2 | 1.70 | 0.77 | 1 | 62 | 25.5 | 1 | 0 | 1 | 1 | 0 | 0 | 0 | Stable angina |
| 7 | LAD | 40 | 0.89 | 11.1 | 5.12 | 0.17 | 1 | 82 | 31.4 | 1 | 1 | 1 | 1 | 0 | 0 | 0 | Stable angina |
| 8 | LAD | 44 | 0.88 | 22.9 | 2.49 | 0.54 | 1 | 62 | 36.7 | 1 | 1 | 1 | 1 | 0 | 0 | 0 | Stable angina |
| 9 | LAD | 25 | 0.89 | 10.4 | 1.98 | 0.11 | 0 | 59 | 32.0 | 1 | 2 | 0 | 1 | 0 | 1 | 0 | Stable angina |
| 10 | RCA | 32 | 0.88 | 11.0 | 6.39 | 0.14 | 1 | 73 | 24.9 | 1 | 0 | 1 | 1 | 0 | 0 | 0 | Stable angina |
| 11 | RCA | 42 | 0.86 | 12.2 | 2.23 | 0.26 | 1 | 75 | 28.3 | 1 | 0 | 1 | 0 | 0 | 1 | 0 | Stable angina |
| 12 | RCA | 27 | 0.95 | 7.6 | 6.37 | 0.42 | 1 | 61 | 28.1 | 1 | 1 | 1 | 0 | 1 | 1 | 0 | Stable angina |
| 13 | LAD | 42 | 0.83 | 11.9 | 3.98 | 0.23 | 1 | 54 | 34.6 | 1 | 1 | 1 | 1 | 1 | 1 | 0 | Stable angina |
| 14 | LCx | 38 | 0.86 | 15.4 | 1.25 | 0.25 | 1 | 77 | 28.1 | 1 | 1 | 1 | 1 | 1 | 1 | 0 | Stable angina |
| 15 | LAD | 34 | 0.88 | 11.5 | 1.68 | 0.13 | 1 | 40 | 24.0 | 1 | 2 | 1 | 1 | 1 | 1 | 0 | Silent ischemia |
| 16 | LAD | 44 | 0.75 | 13.8 | 1.54 | 0.22 | 1 | 66 | 28.7 | 0 | 2 | 1 | 1 | 0 | 0 | 0 | Silent ischemia |
| 17 | LAD | 42 | 0.79 | 11.8 | 0.52 | 0.03 | 0 | 68 | 26.8 | 1 | 1 | 1 | 1 | 1 | 1 | 0 | Stable angina |
| 18 | LAD | 53 | 0.76 | 14.2 | 1.10 | 0.45 | 1 | 72 | 22.9 | 1 | 1 | 1 | 1 | 1 | 1 | 0 | Silent ischemia |
| 19 | LCx | 39 | 0.80 | 9.3 | 1.78 | 0.29 | 1 | 83 | 18.6 | 1 | 1 | 1 | 1 | 1 | 1 | 0 | Stable angina |
| 20 | LAD | 44 | 0.80 | 12.6 | 1.68 | 0.16 | 1 | 83 | 21.3 | 0 | 1 | 0 | 0 | 0 | 0 | 0 | Other |
| 21 | LAD | 44 | 0.62 | 11.4 | 1.23 | 0.26 | 1 | 77 | 24.5 | 1 | 1 | 1 | 1 | 1 | 1 | 0 | Silent ischemia |
| 22 | LCx | 50 | 0.80 | 14.4 | 0.76 | 0.34 | 1 | 69 | 30.9 | 1 | 1 | 1 | 1 | 1 | 1 | 0 | Silent ischemia |
| 23 | RCA | 32 | 0.96 | 14.8 | 1.00 | 0.86 | 0 | 74 | 20.4 | 0 | 1 | 0 | 1 | 0 | 0 | 0 | Silent ischemia |
| 24 | LCx | 38 | 0.93 | 10.8 | 1.76 | 0.08 | 1 | 67 | 24.2 | 0 | 1 | 1 | 1 | 1 | 1 | 0 | Silent ischemia |
| 25 | RCA | 35 | 0.94 | 11.1 | 2.43 | 0.13 | 1 | 65 | 22.3 | 0 | 1 | 1 | 1 | 1 | 1 | 0 | Silent ischemia |

ACS, acute coronary syndrome; AMI, acute myocardial infarction; BMI, body mass index; CABG, coronary artery bypass grafting; DS%, percent diameter stenosis; LAD, left anterior descending artery; LCR, lipid-to-cap ratio; LCx, left circumflex; MLA, minimal lumen area; OCT, optical coherence tomography; PCI, percutaneous coronary intervention; QCA, quantitative coronary angiography; RCA, right coronary artery; RWS, radial wall strain; μFR, Murray law-based quantitative flow ratio.

**^*^** For smoking, 2 indicates current, 1 indicates previous, 0 indicates never.

# **Supplemental Table 3.** List of participating centers

| **Participating centers** | **Number of participants** |
| --- | --- |
| Public Health Clinical Center of Chengdu, Chengdu | 1 |
| Dachuan District People's Hospital of Dazhou City, Dazhou | 2 |
| Fujian Medical University Union Hospital, Fuzhou | 3 |
| Fudan University Zhongshan Hospital, Shanghai | 19 |
| Guangdong Provincial People’s Hospital, Guangzhou | 2 |
| Guiyang Huaxi District People’s Hospital, Guiyang | 2 |
| Nayong County People's Hospital, Bijie | 1 |
| Pingtang County People’s Hospital, Guizhou | 1 |
| Qingzhen First People’s Hospital, Guiyang | 1 |
| Sansui County People’s Hospital, Guizhou | 1 |
| Xifeng County People’s Hospital, Guiyang | 1 |
| Xing Yi People’s Hospital, Xingyi | 1 |
| The Second Affiliated Hospital of Guizhou Medical University, Kaili | 1 |
| The Affiliated Hospital of Guizhou Medical University, Guiyang | 8 |
| The Third Bethune Hospital of Jilin University, Changchun | 1 |
| The People’s Hospital of Liaoning Province, Shenyang | 1 |
| The First Affiliated Hospital of Nanchang University, Nanchang | 1 |
| Panzhou People’s Hospital, Panzhou | 1 |
| RongJiang County People’s Hospital, Guizhou | 1 |
| Shanghai General Hospital, Shanghai | 2 |
| Shanghai Chest Hospital, Shanghai | 1 |
| West China Hospital Sichuan University, Chengdu | 5 |
| Zhongnan Hospital of Wuhan University, Wuhan | 1 |
| The First Affiliated Hospital of Xi’an JiaoTong University, Xi’an | 1 |
| Xinyang Central Hospital, Xinyang | 1 |
| The Affiliated Hospital of Xuzhou Medical University, Xuzhou | 14 |
| The Third People’s Hospital of Yibin, Yibin | 1 |
| The 7^th^ People’s Hospital of Zhengzhou, Zhengzhou | 10 |
| The First Affiliated Hospital of Chongqing Medical University, Chongqing | 1 |
| The Fifth Affiliated Hospital of Zunyi Medical University, Zhuhai | 1 |

# **Supplemental Table 4.** STROBE checklist

|  | Item No | Recommendation | Reported on page |
| --- | --- | --- | --- |
| **Title and abstract** | 1 | (*a*) Indicate the study’s design with a commonly used term in the title or the abstract | 1 |
|  |  | (*b*) Provide in the abstract an informative and balanced summary of what was done and what was found | 1 |
| Introduction | | |  |
| Background/rationale | 2 | Explain the scientific background and rationale for the investigation being reported | 5-6 |
| Objectives | 3 | State specific objectives, including any prespecified hypotheses | 6-7 |
| Methods | | |  |
| Study design | 4 | Present key elements of study design early in the paper | 7 |
| Setting | 5 | Describe the setting, locations, and relevant dates, including periods of recruitment, exposure, follow-up, and data collection | 7-11 |
| Participants | 6 | (*a*) Give the eligibility criteria, and the sources and methods of selection of participants | 7-8 |
| Variables | 7 | Clearly define all outcomes, exposures, predictors, potential confounders, and effect modifiers. Give diagnostic criteria, if applicable | 7-11 |
| Data sources/ measurement | 8* | For each variable of interest, give sources of data and details of methods of assessment (measurement). Describe comparability of assessment methods if there is more than one group | 8-11 |
| Bias | 9 | Describe any efforts to address potential sources of bias | 8 |
| Study size | 10 | Explain how the study size was arrived at | 8 |
| Quantitative variables | 11 | Explain how quantitative variables were handled in the analyses. If applicable, describe which groupings were chosen and why | 9-11 |
| Statistical methods | 12 | (*a*) Describe all statistical methods, including those used to control for confounding | 12 |
|  |  | (*b*) Describe any methods used to examine subgroups and interactions | 12 |
|  |  | (*c*) Explain how missing data were addressed | 8 |
|  |  | (*d*) If applicable, describe analytical methods taking account of sampling strategy | Not applicable |
|  |  | (*e*) Describe any sensitivity analyses | 12 |
| Results | | |  |
| Participants | 13* | (a) Report numbers of individuals at each stage of study—eg numbers potentially eligible, examined for eligibility, confirmed eligible, included in the study, completing follow-up, and analysed | 13 |
|  |  | (b) Give reasons for non-participation at each stage | 13 |
|  |  | (c) Consider use of a flow diagram | Figure 1 |
| Descriptive data | 14* | (a) Give characteristics of study participants (eg demographic, clinical, social) and information on exposures and potential confounders | 13, Table 1, Supplemental Table 2 |
|  |  | (b) Indicate number of participants with missing data for each variable of interest | 13 |
| Outcome data | 15* | Report numbers of outcome events or summary measures | 13-14 |
| Main results | 16 | (*a*) Give unadjusted estimates and, if applicable, confounder-adjusted estimates and their precision (eg, 95% confidence interval). Make clear which confounders were adjusted for and why they were included | 13-14 |
|  |  | (*b*) Report category boundaries when continuous variables were categorized | 13-14 |
|  |  | (*c*) If relevant, consider translating estimates of relative risk into absolute risk for a meaningful time period | Not applicable |
| Other analyses | 17 | Report other analyses done—eg analyses of subgroups and interactions, and sensitivity analyses | 15-17 |
| Discussion | | |  |
| Key results | 18 | Summarise key results with reference to study objectives | 17 |
| Limitations | 19 | Discuss limitations of the study, taking into account sources of potential bias or imprecision. Discuss both direction and magnitude of any potential bias | 22 |
| Interpretation | 20 | Give a cautious overall interpretation of results considering objectives, limitations, multiplicity of analyses, results from similar studies, and other relevant evidence | 17-22 |
| Generalisability | 21 | Discuss the generalisability (external validity) of the study results | 22 |
| Other information | | |  |
| Funding | 22 | Give the source of funding and the role of the funders for the present study and, if applicable, for the original study on which the present article is based | 24 |

*Give information separately for exposed and unexposed groups.

# **Supplemental Table 5.** Overall patterns of Decision I to Decision IV

| **Baseline information** | | | | | | | **Decision I, %** | | | **Decision II, %** | | | **Decision III, %** | | | | **Decision IV, %** | | | |
| --- | --- | --- | --- | --- | --- | --- | --- | --- | --- | --- | --- | --- | --- | --- | --- | --- | --- | --- | --- | --- |
| **No.** | **Vessel** | **QCA-DS%** | **μFR** | **RWS_max_, %** | **OCT-MLA, mm^2^** | **LCR** | **Standard medical therapy** | **PCI** | **CABG** | **Standard medical therapy** | **PCI** | **CABG** | **Standard medical therapy** | **Intensive medical therapy** | **PCI** | **CABG** | **Standard medical therapy** | **Intensive medical therapy** | **PCI** | **CABG** |
| 1 | RCA | 49 | 0.82 | 19.6 | 1.25 | 0.80 | 37.2 | 62.8 | 0.0 | 84.9 | 15.1 | 0.0 | 5.8 | 40.7 | 53.5 | 0.0 | 0.0 | 14.0 | 86.0 | 0.0 |
| 2 | LAD | 47 | 0.81 | 10.1 | 1.78 | 0.59 | 43.5 | 56.5 | 0.0 | 81.2 | 18.8 | 0.0 | 21.2 | 65.9 | 12.9 | 0.0 | 10.6 | 20.0 | 69.4 | 0.0 |
| 3 | RCA | 42 | 0.91 | 13.7 | 2.32 | 0.59 | 85.4 | 14.6 | 0.0 | 96.3 | 3.7 | 0.0 | 19.5 | 56.1 | 24.4 | 0.0 | 13.4 | 57.3 | 29.3 | 0.0 |
| 4 | RCA | 34 | 0.94 | 20.9 | 1.95 | 0.47 | 92.6 | 7.4 | 0.0 | 100.0 | 0.0 | 0.0 | 17.3 | 54.3 | 28.4 | 0.0 | 2.5 | 39.5 | 58.0 | 0.0 |
| 5 | RCA | 46 | 0.92 | 9.3 | 2.78 | 0.09 | 35.4 | 64.6 | 0.0 | 80.5 | 19.5 | 0.0 | 34.1 | 47.6 | 18.3 | 0.0 | 15.9 | 42.7 | 41.5 | 0.0 |
| 6 | RCA | 45 | 0.85 | 21.2 | 1.70 | 0.77 | 68.7 | 30.1 | 1.2 | 91.6 | 8.4 | 0.0 | 8.4 | 45.8 | 45.8 | 0.0 | 1.2 | 24.1 | 74.7 | 0.0 |
| 7 | LAD | 40 | 0.89 | 11.1 | 5.12 | 0.17 | 97.5 | 2.5 | 0.0 | 98.8 | 1.2 | 0.0 | 35.8 | 64.2 | 0.0 | 0.0 | 35.8 | 63.0 | 1.2 | 0.0 |
| 8 | LAD | 44 | 0.88 | 22.9 | 2.49 | 0.54 | 38.0 | 62.0 | 0.0 | 78.5 | 21.5 | 0.0 | 3.8 | 38.0 | 58.2 | 0.0 | 2.5 | 43.0 | 54.4 | 0.0 |
| 9 | LAD | 25 | 0.89 | 10.4 | 1.98 | 0.11 | 98.7 | 1.3 | 0.0 | 98.7 | 1.3 | 0.0 | 42.3 | 57.7 | 0.0 | 0.0 | 19.2 | 38.5 | 42.3 | 0.0 |
| 10 | RCA | 32 | 0.88 | 11.0 | 6.39 | 0.14 | 97.4 | 2.6 | 0.0 | 98.7 | 1.3 | 0.0 | 43.6 | 56.4 | 0.0 | 0.0 | 42.3 | 57.7 | 0.0 | 0.0 |
| 11 | RCA | 42 | 0.86 | 12.2 | 2.23 | 0.26 | 72.0 | 26.7 | 1.3 | 94.7 | 5.3 | 0.0 | 22.7 | 66.7 | 10.7 | 0.0 | 20.0 | 53.3 | 26.7 | 0.0 |
| 12 | RCA | 27 | 0.95 | 7.6 | 6.37 | 0.42 | 94.8 | 5.2 | 0.0 | 98.7 | 1.3 | 0.0 | 57.1 | 39.0 | 3.9 | 0.0 | 50.6 | 48.1 | 1.3 | 0.0 |
| 13 | LAD | 42 | 0.83 | 11.9 | 3.98 | 0.23 | 83.3 | 16.7 | 0.0 | 93.6 | 6.4 | 0.0 | 35.9 | 59.0 | 5.1 | 0.0 | 26.9 | 62.8 | 10.3 | 0.0 |
| 14 | LCx | 38 | 0.86 | 15.4 | 1.25 | 0.25 | 96.1 | 3.9 | 0.0 | 97.4 | 2.6 | 0.0 | 18.2 | 54.5 | 27.3 | 0.0 | 3.9 | 19.5 | 76.6 | 0.0 |
| 15 | LAD | 34 | 0.88 | 11.5 | 1.68 | 0.13 | 79.2 | 20.8 | 0.0 | 94.8 | 5.2 | 0.0 | 39.0 | 53.2 | 7.8 | 0.0 | 7.8 | 23.4 | 68.8 | 0.0 |
| 16 | LAD | 44 | 0.75 | 13.8 | 1.54 | 0.22 | 38.5 | 60.3 | 1.3 | 9.0 | 89.7 | 1.3 | 1.3 | 16.7 | 80.8 | 1.3 | 0.0 | 5.1 | 93.6 | 1.3 |
| 17 | LAD | 42 | 0.79 | 11.8 | 0.52 | 0.03 | 74.4 | 25.6 | 0.0 | 59.0 | 41.0 | 0.0 | 20.5 | 64.1 | 15.4 | 0.0 | 2.6 | 11.5 | 85.9 | 0.0 |
| 18 | LAD | 53 | 0.76 | 14.2 | 1.10 | 0.45 | 14.3 | 84.4 | 1.3 | 11.7 | 87.0 | 1.3 | 0.0 | 9.1 | 89.6 | 1.3 | 0.0 | 2.6 | 96.1 | 1.3 |
| 19 | LCx | 39 | 0.80 | 9.3 | 1.78 | 0.29 | 46.2 | 52.6 | 1.3 | 52.6 | 46.2 | 1.3 | 23.1 | 43.6 | 32.1 | 1.3 | 9.0 | 24.4 | 65.4 | 1.3 |
| 20 | LAD | 44 | 0.80 | 12.6 | 1.68 | 0.16 | 56.4 | 43.6 | 0.0 | 61.5 | 38.5 | 0.0 | 14.1 | 41.0 | 44.9 | 0.0 | 5.1 | 24.4 | 70.5 | 0.0 |
| 21 | LAD | 44 | 0.62 | 11.4 | 1.23 | 0.26 | 56.4 | 42.3 | 1.3 | 14.1 | 83.3 | 2.6 | 7.7 | 30.8 | 59.0 | 2.6 | 2.6 | 11.5 | 84.6 | 1.3 |
| 22 | LCx | 50 | 0.80 | 14.4 | 0.76 | 0.34 | 12.8 | 87.2 | 0.0 | 35.9 | 64.1 | 0.0 | 2.6 | 17.9 | 79.5 | 0.0 | 0.0 | 3.8 | 93.6 | 2.6 |
| 23 | RCA | 32 | 0.96 | 14.8 | 1.00 | 0.86 | 88.3 | 11.7 | 0.0 | 98.7 | 1.3 | 0.0 | 22.1 | 54.5 | 23.4 | 0.0 | 7.8 | 39.0 | 53.2 | 0.0 |
| 24 | LCx | 38 | 0.93 | 10.8 | 1.76 | 0.08 | 88.3 | 11.7 | 0.0 | 96.1 | 3.9 | 0.0 | 44.2 | 50.6 | 5.2 | 0.0 | 22.1 | 31.2 | 46.8 | 0.0 |
| 25 | RCA | 35 | 0.94 | 11.1 | 2.43 | 0.13 | 31.2 | 68.8 | 0.0 | 80.5 | 19.5 | 0.0 | 28.6 | 50.6 | 20.8 | 0.0 | 19.5 | 49.4 | 31.2 | 0.0 |

Derived from a total of 1975 lesion-based evaluations. CABG, coronary artery bypass grafting; DS%, percent diameter stenosis; LAD, left anterior descending artery; LCx, left circumflex; MLA, minimal lumen area; OCT, optical coherence tomography; PCI, percutaneous coronary intervention; QCA, quantitative coronary angiography; RCA, right coronary artery; RWS, radial wall strain; μFR, Murray law-based quantitative flow ratio.

# **Supplemental Table 6.** Predictors of revascularization decision change

|  | **Univariate** | | **Multivariate** | |
| --- | --- | --- | --- | --- |
|  | **OR (95% CI)** | **P value** | **OR (95% CI)** | **P value** |
| Age >40 y | 1.05 (0.67, 1.65) | 0.828 | / | / |
| Experience in interventional cardiology (≤2 y as reference) | | | | |
| >2 and ≤5 y | 0.44 (0.18, 1.05) | 0.065 | 0.41 (0.17, 0.97) | 0.042 |
| >5 and ≤10 y | 0.41 (0.17, 0.99) | 0.047 | 0.42 (0.17, 1.01) | 0.051 |
| >10 y | 0.40 (0.17, 0.89) | 0.026 | 0.39 (0.17, 0.90) | 0.026 |
| Number of yearly PCI (≤100 as reference) | | | | |
| 101-300 | 0.96 (0.49, 1.87) | 0.897 | / | / |
| 301-500 | 0.78 (0.39, 1.56) | 0.480 | / | / |
| >500 | 0.72 (0.36, 1.43) | 0.345 | / | / |
| Experience with FFR (no experience as reference) | | | | |
| <1 y | 0.63 (0.17, 2.29) | 0.479 | / | / |
| 1-3 y | 0.68 (0.20, 2.32) | 0.536 | / | / |
| >3 y | 0.63 (0.19, 2.06) | 0.446 | / | / |
| Experience with μFR (no experience as reference) | | | | |
| <1 y | 1.58 (0.86, 2.90) | 0.142 | 1.54 (0.86, 2.75) | 0.143 |
| 1-3 y | 1.73 (0.93, 3.22) | 0.081 | 1.79 (0.97, 3.28) | 0.061 |
| >3 y | 1.28 (0.70, 2.36) | 0.421 | 1.41 (0.77, 2.59) | 0.266 |
| Experience with IVUS (<1 y as reference) | | | | |
| 1-3 y | 0.91 (0.29, 2.88) | 0.873 | / | / |
| >3 y | 0.82 (0.29, 2.32) | 0.713 | / | / |
| Experience with OCT (no experience as reference) | | | | |
| <1 y | 1.21 (0.55, 2.67) | 0.636 | / | / |
| 1-3 y | 1.21 (0.50, 2.90) | 0.677 | / | / |
| >3 y | 1.03 (0.44, 2.42) | 0.950 | / | / |
| Experience with RWS (no experience as reference) | | | | |
| <1 y | 0.97 (0.56, 1.71) | 0.885 | / | / |
| ≥1 y | 1.43 (0.69, 2.99) | 0.334 | / | / |

FFR, fractional flow reserve; IVUS, intravascular ultrasound; OCT, optical coherence tomography; OR, odds ratio; PCI, percutaneous coronary intervention; RWS, radial wall strain; μFR, Murray law-based quantitative flow ratio.

# **Supplemental Figure 1.** Influence of μFR on revascularization decision making


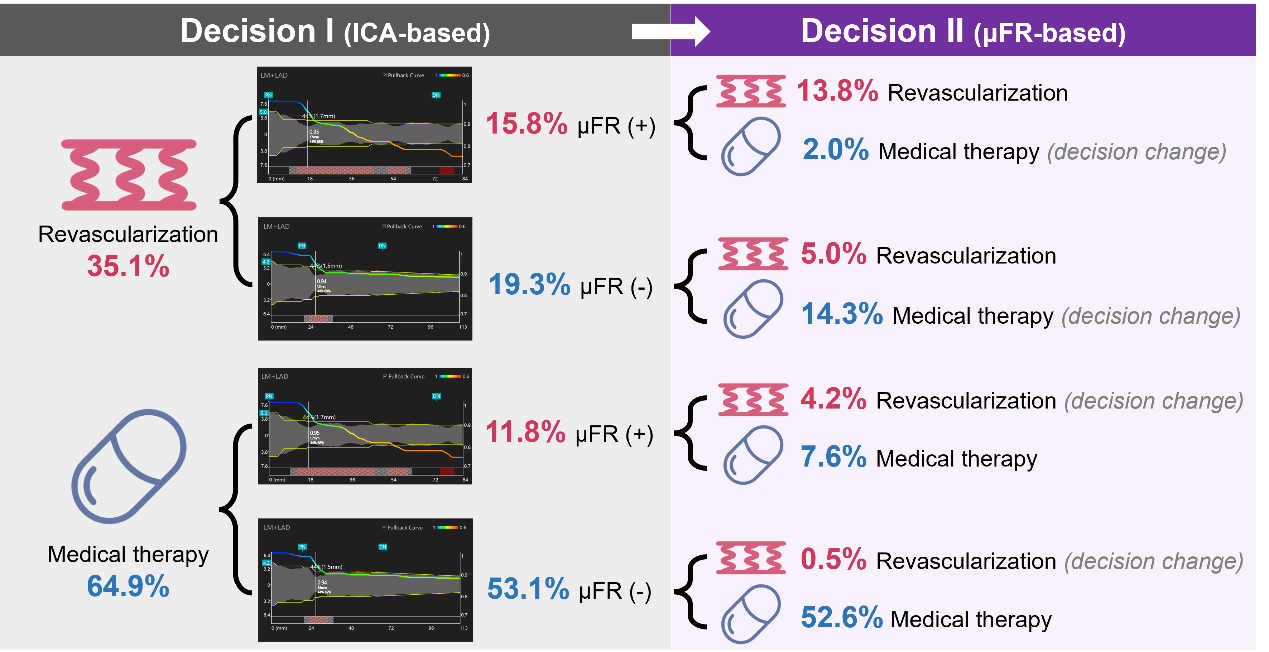


In the initial ICA-based Decision I, 35.1% (693/1975) of cases were recommended for revascularization, while 64.9% (1282/1975) for medical therapy alone. However, 31.1% (614/1975) of these decisions were discordant with dichotomous μFR values: 19.3% (381/1975) planned for revascularization was physiologically nonsignificant (μFR >0.80), and 11.8% (233/1975) planned for medical therapy was physiological significant (μFR ≤0.80). Following μFR disclosure, 21.1% (416/1975) of the initial ICA-based decisions were revised in Decision II, mainly (14.3% [282/1975]) shifting from revascularization to medical therapy due to negative μFR. Despite this adjustment, 15.2% (300/1975) of Decision II remained discordant with μFR: 5.6% (110/1975) were still planned for revascularization despite having a μFR >0.80, and 9.6% (190/1975) were planned for medical therapy despite having a positive μFR.

ICA, invasive coronary angiography; μFR, Murray law-based quantitative flow ratio.

# **Supplemental Figure 2.** Influence of RWS on revascularization decision making


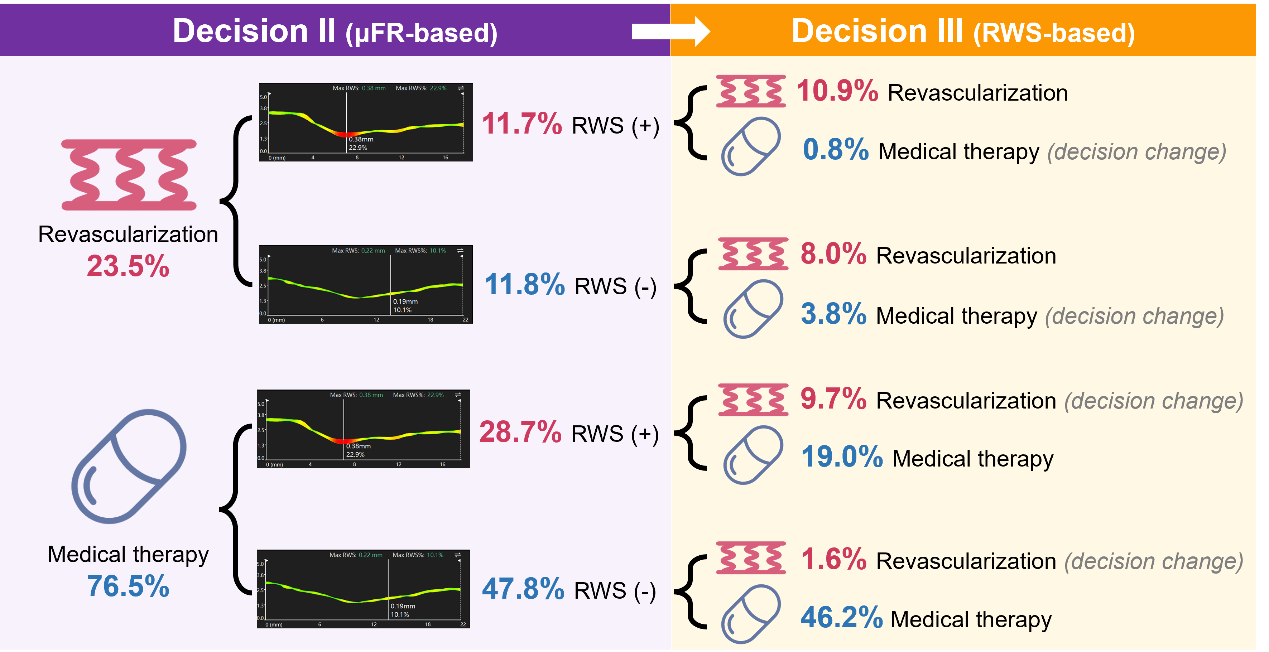


The introduction of RWS led to further changes in revascularization decision for 15.9% (315/1975) of cases from Decision II to Decision III. Among the 1510 (76.5%) cases initially planned for medical therapy alone in Decision II, 566 (28.7%) cases had a positive RWS (≥13.0%), with 192 (9.7%) of these being redesignated to revascularization; the remaining 944 (47.8%) cases with a negative RWS (<13.0%) saw only 31 (1.6%) such shifts. Comparatively, among 465 (23.5%) cases initially planned for revascularization in Decision II, 233 (11.8%) cases had a negative RWS, and 76 (3.8%) of these were shifted to medical therapy alone; while only 16 (0.8%) of the remaining 232 (11.7%) cases with a positive RWS underwent such changes.

RWS, radial wall strain; μFR, Murray law-based quantitative flow ratio.

# **Supplemental Figure 3.** Step-wise change of revascularization decision patterns


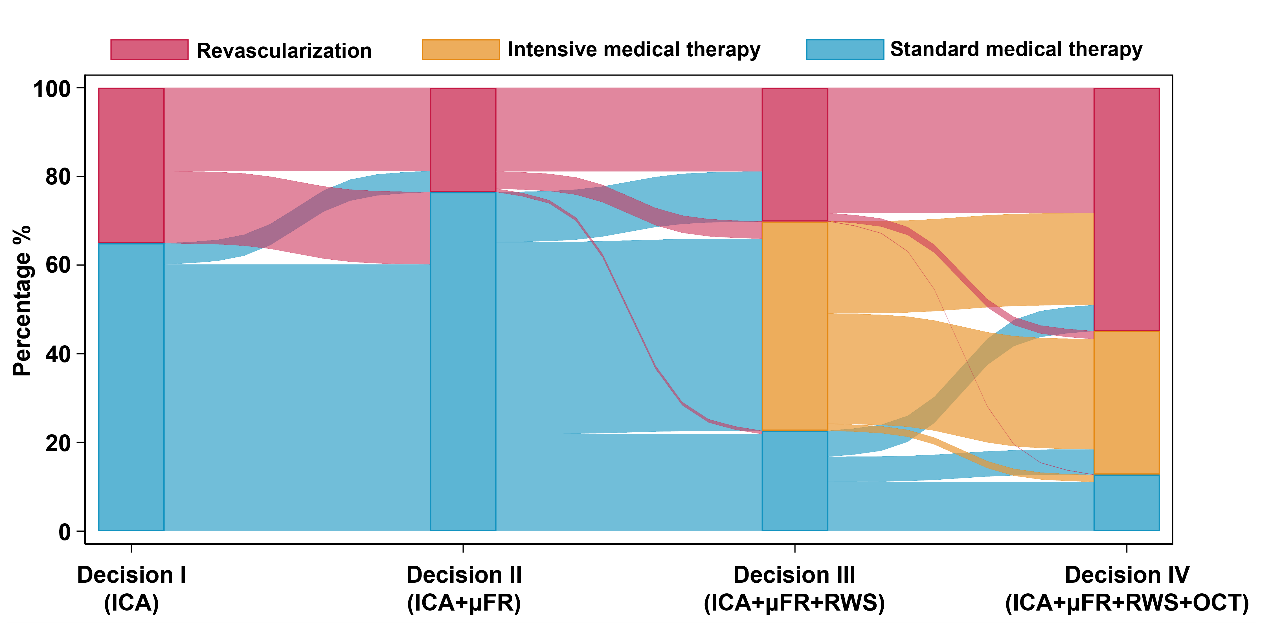


Compared with ICA-based Decision I, the introduction of μFR resulted in a decision change in 21.1% (416/1975) of cases (p<0.0001), including 16.3% (322/1975) shifting from revascularization to medical therapy alone and 4.8% (94/1975) from medical therapy alone to revascularization. Subsequently, after RWS was disclosed, the revascularization decision changed further in 15.9% (315/1975) of cases (p<0.0001), with 11.3% (223/1975) changing from medical therapy alone to revascularization and 4.7% (92/1975) from revascularization to medical therapy alone. 855 (43.3%) cases initially assigned to standard medical therapy in Decision II were escalated to intensive medical therapy in Decision III. Finally, after OCT was disclosed, the revascularization decisions changed in 28.6% (564/1975) of cases (p<0.0001), with the majority (26.6% [526/1975]) involving a shift from medical therapy alone to revascularization. Additionally, 5.7% (112/1975) of cases initially planned for standard medical therapy in Decision III were escalated to intensive medical therapy in Decision IV.

ICA, invasive coronary angiography; OCT, optical coherence tomography; RWS, radial wall strain; μFR, Murray law-based quantitative flow ratio.

# **Supplemental Figure 4.** Influence of OCT on revascularization decision making


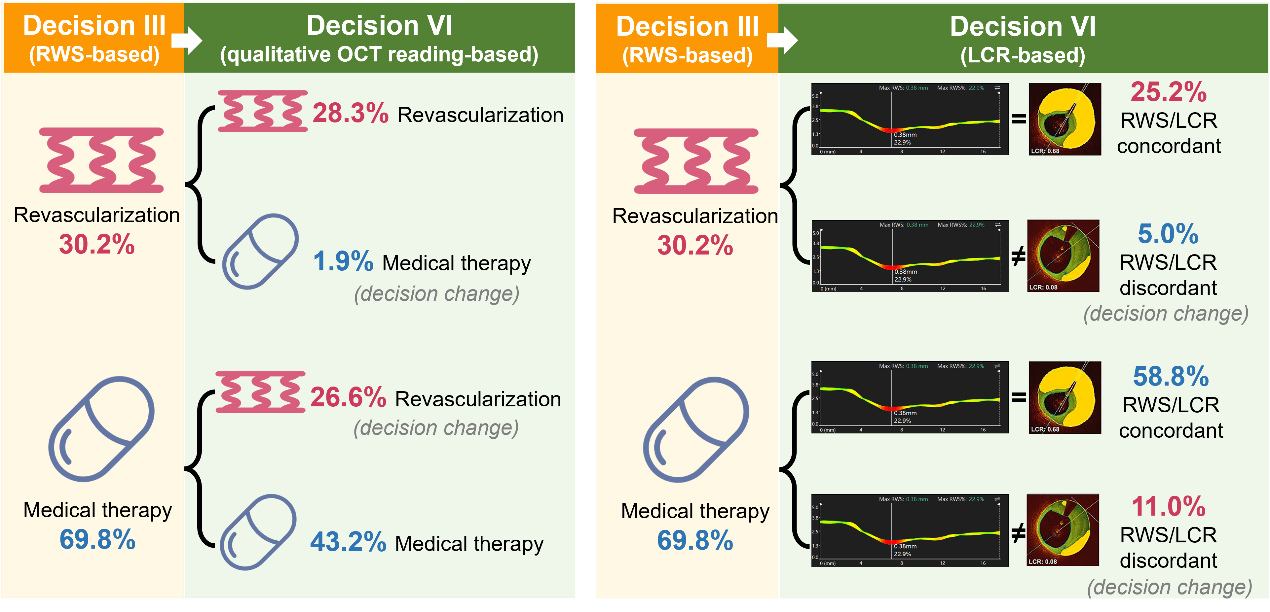


The disclosure of OCT pullback data and MLA further influenced revascularization decisions in 28.6% (564/1975) of cases from Decision III to Decision IV. Specifically, 26.6% (526/1379) of cases shifted from medical therapy alone to revascularization, while 1.9% (38/1975) shifted from revascularization to medical therapy alone. Notably, if Decision IV had been strictly guided by LCR rather than qualitative OCT interpretation, the RWS-based Decision III would have been altered in only 16.1% (317/1975) of cases. RWS and LCR were concordant when both were positive (RWS ≥13.0% and LCR >0.33) or negative (RWS <13.0% and LCR ≤0.33); while RWS and LCR were discordant if RWS ≥13.0% and LCR ≤0.33 and if RWS <13.0% and LCR >0.33.

OCT, optical coherence tomography; RWS, radial wall strain; LCR, lipid-to-cap ratio.
